# Restoration of dendritic cell homeostasis and Type I/Type III interferon levels in convalescent COVID-19 individuals

**DOI:** 10.1186/s12865-022-00526-z

**Published:** 2022-10-26

**Authors:** Anuradha Rajamanickam, Nathella Pavan Kumar, Arul Nancy Pandiaraj, Nandhini Selvaraj, Saravanan Munisankar, Rachel Mariam Renji, Vijayalakshmi Venkatramani, Manoj Murhekar, Jeromie Wesley Vivian Thangaraj, Muthusamy Santhosh Kumar, Chethrapilly Purushothaman Girish Kumar, Tarun Bhatnagar, Manickam Ponnaiah, Ramasamy Sabarinathan, Velusamy Saravanakumar, Subash Babu

**Affiliations:** 1 ICER-ICMR-NIRT-International Center for Excellence in Research, Chennai, Tamil Nadu India; 2 Immunology-ICMR-National Institute for Research in Tuberculosis, Chennai, Tamil Nadu India; 3 ICMR-National Institute of Epidemiology, Chennai, Tamil Nadu India

**Keywords:** Dendritic cell subsets, pDC, mDC, Type I IFNs, Type III IFNs, COVID-19, Acute and convalescent COVID-19

## Abstract

**Background:**

Plasmacytoid and myeloid dendritic cells play a vital role in the protection against viral infections. In COVID-19, there is an impairment of dendritic cell (DC) function and interferon secretion which has been correlated with disease severity.

**Results:**

In this study, we described the frequency of DC subsets and the plasma levels of Type I (IFNα, IFNβ) and Type III Interferons (IFNλ1), IFNλ2) and IFNλ3) in seven groups of COVID-19 individuals, classified based on days since RT-PCR confirmation of SARS-CoV2 infection. Our data shows that the frequencies of pDC and mDC increase from Days 15–30 to Days 61–90 and plateau thereafter. Similarly, the levels of IFNα, IFNβ, IFNλ1, IFNλ2 and IFNλ3 increase from Days 15–30 to Days 61–90 and plateau thereafter. COVID-19 patients with severe disease exhibit diminished frequencies of pDC and mDC and decreased levels of IFNα, IFNβ, IFNλ1, IFNλ2 and IFNλ3. Finally, the percentages of DC subsets positively correlated with the levels of Type I and Type III IFNs.

**Conclusion:**

Thus, our study provides evidence of restoration of homeostatic levels in DC subset frequencies and circulating levels of Type I and Type III IFNs in convalescent COVID-19 individuals.

**Supplementary Information:**

The online version contains supplementary material available at 10.1186/s12865-022-00526-z.

## Background

Among innate immune cells which have a critical role in the anti-SARS-CoV-2 immune response, one of the main players are the dendritic cells (DCs) [1]. SARS-CoV-2 infection induces an impairment in the functions of interferons (IFNs), antigen presentation and decreased numbers of DCs in the peripheral blood [2]. Several reports indicated that the DC numbers were diminished due to infection in peripheral blood and it is associated with the severity of this disease [3, 4]. This decrease could be due to the migration of DC subsets to the lung and other inflammatory loci [5, 6], enhanced apoptosis of DCs and the suppressive consequences of myeloid-derived suppressor cells [7]. Type I FNs play an important role in viral infections [8] and major producers of Type I IFNs have a crucial function in COVID-19. Also, various studies have shown that severe patients were associated with decreased IFN responses [9, 10]. Several reports determined that SARS-CoV-2 affects pDCs by producing IFN-I [7]. However, the function of DC subsets and Type I and III IFNs in acute and convalescent SARS-CoV-2 individuals are not completely explored.

Following COVID-19, the restoration of DC impairment might be essential, because the stabilization of the innate immune system is required for return to homeostasis. This recovery is essential in terms in terms of return to normalcy of innate immune responses as DCs and Type I/III interferons are important in the prolonged protection [11]. Hence, we wanted to study the frequencies of DC subsets and circulating levels of Type I and Type III IFNs in convalescent COVID-19 individuals more than 150 days after infection following RT PCR confirmation.

## Results

### Study population characteristics

Demographics and clinical characteristics of the study population are shown in Tables 1 and 2 as previously described [12–14]. The median age ranges from 36—45.5 years among the groups. In all the groups, the female number was slightly higher than the number of male patients. However, they are not statistically different among the groups. Individuals with Hypertension were 26% in the acute phase group and in the remaining groups i.e., 31–60 days to more than 180 days, the percentage ranged from 18 to 30%. Individuals with Diabetes Mellitus were 19% in the acute phase and in the remaining groups i.e., 31–60 days to more than 180 days, the percentage ranged from 19 to 30%. Individuals with Asthma were 5% in the acute phase and in the remaining groups i.e., 31–60 days (6%), 61–90 days (3%), 91–120 days (3%) and 151–180 days (3%). The other clinical symptoms such as fever, cough, chills, sore throat, runny nose, loss of taste and smell, muscle aches, joint pain, abdominal pain, vomit, and diarrhoea were present almost in all the groups. Individuals with seizures were 3% in 31–60 days alone and Neuro related disorders were 5% in the 61–90 days group alone. 3% of chronic kidney diseases were in 91–120 days and more than 180 groups of individuals. 15–30 days (6%), 31–60 days (3%) and 61–90 days (3%), 7% of the severely infected were with heart diseases. Individuals with rheumatic fever were 3% in 61–90 days and 151–180 days (3%). Individuals treated with corticosteroids were 15–30 days (9%), 31–60 days (9%) and 61–90 days (5%), 91–120 days (10%), 121–150 days (3%) and 151–180 days (3%) and severe (20%). Individuals treated with antiviral drugs were 15–30 days (9%), 31–60 days (15%) and 61–90 days (5%), and 91–120 days (13%) and severe (27). Among the severely infected group, 40% of them required hospitalization. We wanted to examine the Type1 and Type III IFN kinetics following RT-PCR confirmation from days 15–30 to more than 180 days. To this end, we combined all the groups irrespective of their disease status (mild, moderate, severe and convalescence) [12–15].

### Increased frequencies of dendritic cell subsets and circulating levels of Type I and Type III IFNs in convalescent COVID-19 individuals over time

**Fig. 1:**
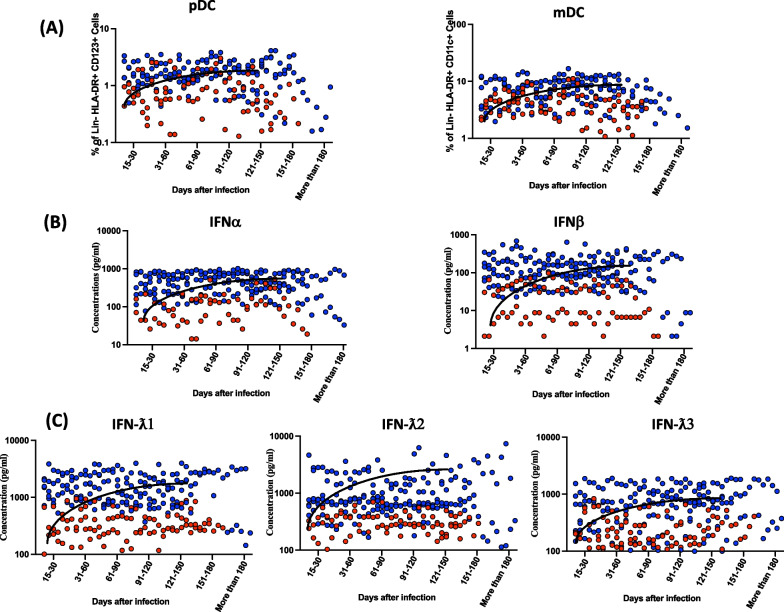
Increased frequencies of dendritic cell subsets and circulating levels of Type I and Type III IFNs in convalescent COVID-19 individuals over time. **A** Analysis of DC subsets from acute and convalescent COVID-19 individuals classified as groups based on days since RT-PCR confirmation. The frequencies of DC subsets (pDC and mDC) are shown with a preferred model for the best fit curve. **B** Analysis of Type I Interferons (IFNα and IFNβ) from acute and convalescent COVID-19 individuals classified as groups based on days since RT-PCR confirmation. The circulating levels of IFNα and IFNβ are shown with a preferred model for the best fit curve (**C).** Analysis of Type III Interferons (IFNλ1, IFNλ2 and IFNλ3) from convalescent COVID-19 individuals classified as groups based on days since RT-PCR confirmation. The circulating levels of IFNλ1, IFNλ2 and IFNλ3 were shown with a preferred model for the best fit curve. The blue colour dot represents mild and the red colour dot represents severely infected individuals. Each dot represents a single individual. The thick black line represents the best fit curve

To determine the frequencies and distribution of dendritic cell subsets in convalescent COVID-19 individuals over time, we assessed the *ex-vivo* frequencies of dendritic cell subsets (pDC and mDC) in the seven groups of COVID-19 individuals. As illustrated in Fig. 1A, and the gating strategy was shown in supply. Fig. S1 (Additional File 1) the frequencies of pDCs and mDCs started increasing from day 15–30 till 91–120 days (first-order model polynomial model fit curve, intermediate monocytes R = 0.29, non-classical monocytes R = 0.42 by Akaike’s Information Criterion) and plateaued thereafter.

Next, we wanted to examine the levels of Type I and Type III IFNs in convalescent COVID-19 individuals over time, we assessed the plasma levels of Type I (IFNα, IFNβ), Type III (IFNλ1, IFNλ2 and IFNλ3) in the seven groups of COVID-19 individuals. As illustrated in Fig. 1B and C, the cross-sectional analysis demonstrated that the levels of Type I and Type III started increasing from days 15–30 (second-order model polynomial model fit curve, IFNα, R = 0.28, IFNβ, R = 0.34, IFNλ1, R = 0.39, IFNλ2, R = 0.32 and IFNλ3 R = 0.52 by Akaike’s Information Criterion) till 151 days after infection.

**Fig. 2:**
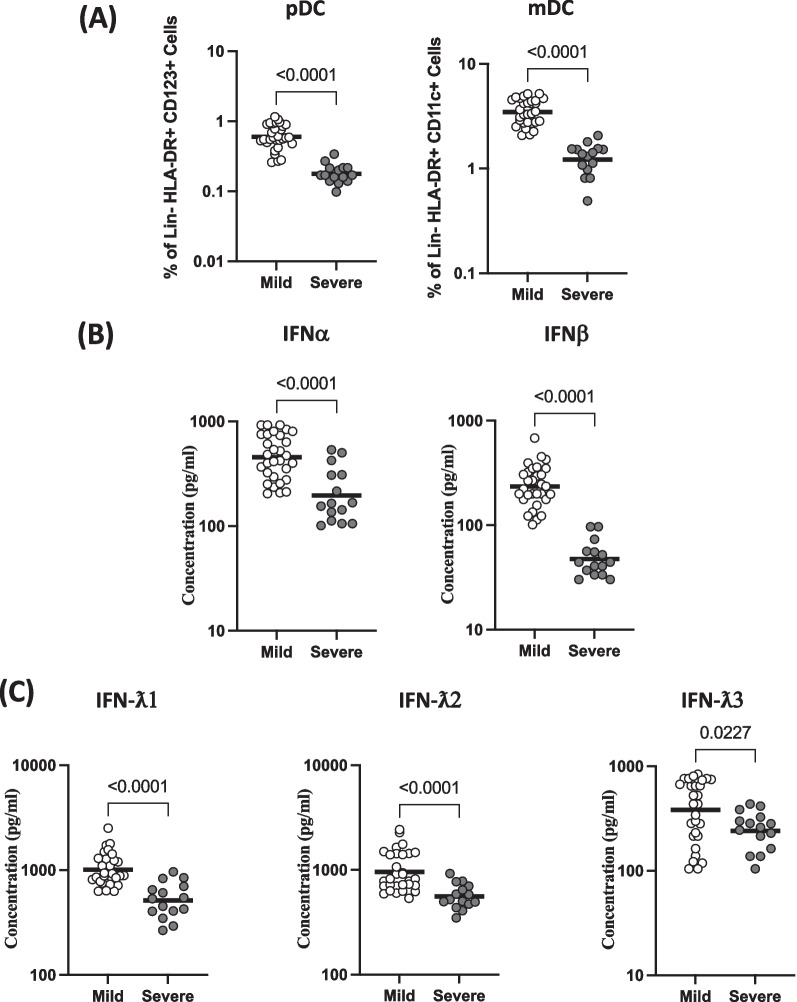
Severe COVID-19 disease is associated with decreased frequencies of DC subsets and circulating levels of Type I and Type III IFN. **A** The frequencies of DC (pDC and mDC) subsets in mild (n = 30) and severe (n = 15) COVID-19 individuals sampled between days 15 to 60 following RT-PCR confirmation. **B** Circulating plasma levels of Type I Interferons (IFNα and IFNβ) in mild (n = 30) and severe (n = 15) COVID-19 individuals sampled between days 15 to 60 following RT-PCR confirmation. **C** Circulating plasma levels of Type III Interferons (IFNλ1, IFNλ2 and IFNλ3) in mild (n = 30) and severe (n = 15) COVID-19 individuals sampled between days 15 to 60 following RT-PCR confirmation. The data are represented as scatter plots with each circle representing a single individual. p values were calculated using the Mann– Whitney U-test

As shown in Additonal file 2: Figure 2, the comparative analysis also exhibited significant differences between seven-time point intervals. Both the DC subsets and the circulating levels of Type 1 and III IFNs showed a gradual and steady increase from days 15–30 to till 150 days and then it started to plateau. The 95% of confidence intervals were shown in Additional file 3: Table 1. Thus, plasma levels of Type I and Type III IFNs are increased over time.

### Severe COVID-19 disease is associated with decreased frequencies of DC subsets and circulating levels of Type I and Type III IFNs

Next, we wanted to determine the frequencies of DC subsets in mild and severely diseased COVID-19 individuals. As shown in Fig. 2A, the frequencies of pDC (GM of 0.60% in mild, 0.18% in severe) and mDC (GM of 3.12% in mild, 0.1% in severe) were significantly lower in severe than the mild COVID-19 individuals. Further, we analysed the effect of COVID-19 disease severity on circulating levels of Type I and Type III IFNs. As shown in Fig. 2B, the levels of IFNα (GM of 456.4 pg/ml in mild, 196.3 pg/ml in severe), IFNβ (GM of 234.7 pg/ml in mild, 47.48 pg/ml in severe), As shown in Fig. 2C, IFNλ1 (GM of 1013 pg/ml in mild, 515 pg/ml in severe), IFNλ2 (GM of 961.7 pg/ml in mild, 559.1 pg/ml in severe) and IFNλ3 (GM of 382 pg/ml in mild, 241 pg/ml in severe) were significantly lower in severe than the mild COVID-19 individuals. Thus, severe COVID-19 disease is associated with decreased frequencies of DC subsets and diminished circulating levels of Type I and Type III IFNs.

### Association between DC subsets and the levels of Type I and Type III IFNs

**Fig. 3:**
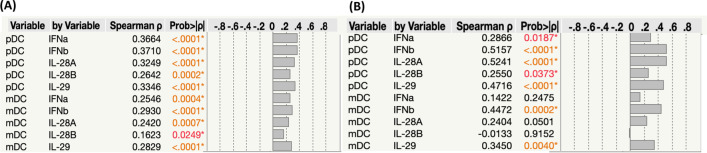
Association between DC subsets and the levels of Type I and Type III IFNs. **A** Correlation analysis between DC subsets Vs Type I and III levels in mild COVID-19 individuals. **B** Correlation analysis between DC subsets Vs Type I and III levels in severe COVID-19 individuals

Next, we wanted to determine the relationship between DC subsets and the levels of Type I and Type III IFNs in mild and severe groups of COVID-19 individuals. As shown in Fig. 3A, mild COVID-19 individuals exhibited a positive correlation between the percentages of DC subsets and the levels of Type I and Type III IFNs. Similarly, severe COVID-19 individuals also exhibited a positive correlation between the percentages of pDC subsets and the levels of Type I and Type III IFNs and the mDCs showed a positive correlation with the levels of IFNβ and IFNλ1 alone (Fig. 3B). Thus, mild and severe COVID-19 individuals exhibited a positive correlation among DC subsets and levels of Type I and Type III IFNs.

## Discussion

DCs play a role in the presentation of antigens, production of cytokines, regulation of inflammatory responses, stimulation of tolerance, immune cell enrolment, and viral spreading [1]. DC frequency and numbers are correlated with the disease severity in COVID-19 [4]. Delayed activation of DC could lead to delayed initiation of adaptive immune response to pathogens. IFN is secreted by diverse cell types and capable of regulating the innate and adaptive immune response [8, 16]. Numerous studies have shown that severe SARS-CoV-2 patients are correlated with lessened numbers of mDCs and pDCs [1, 4, 5]. In our study, both DC subsets started expanding from day 15–30 till 91–120 days and plateaued thereafter. Our study corroborates previous findings that DC subsets were reduced in severe COVID-19 than the mild patients. This decrease could be due to augmented migration from the blood and sequestration in tissues, such as the inflamed lung or lymphoid tissues [17]

Numerous reports determined that Type I and III IFN responses are suppressed during the early phase of the infection in severe COVID-19 patients [2]. pDCs are recognized to play a vital function in the first line of defence in anti-viral simulation, with the capacity to produce IFN-I and III [18, 19]. The reduction of DC subsets could be due to the impairment of myeloid cell functionality [20] and impaired maturation of the DC process that hinders DC homing, and antigen presentation and eventually delay an effective T-cell response [21]. Zhou et al. reported that the capability of DCs to produce pro-inflammatory cytokines and IFN-I was inhibited in COVID-19 patients (Zhou R, Immunity, 2020). An IFN-I deficit supports SARS-CoV-2 to escape leading to the secretion of several ineffective antiviral proinflammatory cytokines which could provoke the cytokine storm [7]. During COVID-19, Type I and III interferon responses were induced in the initial period of infection in regulating the COVID-19 sequence [22]. Individuals with a deficiency in Type I interferon signalling are more susceptible to developing severe COVID-19 (coronavirus disease 2019) disease [23, 24]. Preexisting auto-Abs neutralizing Type I IFNs in APS-1 patients renders them more vulnerable to a massive chance of severe COVID-19 pneumonia across the lifespan [25]. Our data exhibited that expansion of Type I and Type III IFNs from day 15–30 till 120 days and plateaued thereafter. Also, Severe COVID-19 patients exhibited decreased Type I and Type III IFNs. Also, there was a significant positive correlation between the DC subsets and levels of Type I and III IFNs. In this study, we perceived a positive correlation between DC subsets and IFN levels implying that these cell types were the major source of these cytokines as occurs in other viral illnesses [26]. These results corroborated with earlier studies in the SARS-CoV-1 animal models [27] and also with the latest report on the transcriptomic approach [28]. Marongiu et. al. reported that SARS-CoV-2 directly interrelates with conventional DC2s and devices an effective immune escape mechanism that is associated with the severity of disease via downregulating key molecules mandatory for the triggering of T-cells [29]. These data underpin the vital function of IFN-1 secretion in the first line of protection against COVID-19.

Our study has the constraint of not examining the functional effect of these modifications in cellular subsets and IFN pathways. Another limitation is the lack of a healthy control group in our study and that our study was cross-sectional and not longitudinal. We enrolled different individuals in each of the seven categories and did not perform follow-up of the same patients at various time points. However, our study offers an extensive interrogation of DC subsets and levels of IFNs from early to more than 6 months post-infection and disease severity. Our study therefore imply vital changes in DC subsets and IFNs as one of the major occurrence in COVID-19.

## Conclusions

In summary, our study suggests that DC subsets and the levels of Type I and III levels were diminished in the individuals with SARS-CoV-2 at early time points following infection. The DC subsets and the levels of Type I and III were gradually restored more than 6 months after the infection. These results could support a major effect on the type of immune responses in COVID-19. Also, our results imply that Type I and III IFN diminution might be a characteristic feature of COVID-19 and provide a basis for combined therapeutic approaches.

## Methods

### Study population

Acute COVID-19 (15–30 days from RT-PCR confirmation, n = 46) and Convalescent COVID-19 individuals (classified by days after infection as 31–60, n = 33; 61–90, n = 38; 91–120, n = 34; 121–150, n = 32; 151–180, n = 37 and more than 180, n = 40), residing in Chennai and Tiruvallur were enrolled in the study between November 2020 and December 2020 after taking informed consent from the study individuals. This was a cross-sectional study and different individuals were enrolled in the different categories. Those individuals who did not experience any symptoms during the entire course of illness were considered asymptomatic and those who required supplemental oxygen support therapy or those who were admitted to ICU for oxygen support were considered severely ill. Rest were classified under the mild illness category (11–14).

### Haematology

Haematology was performed on all individuals using the Act-5 Diff haematology analyzer (Beckman Coulter). Demographic details and other clinical parameters were shown in Table 1.

### Immunophenotyping and measurement of Type I and III IFNs

Ex vivo phenotyping was done using whole blood. Briefly, to 250 µl aliquots of whole blood, a cocktail of monoclonal antibodies specific for DC subsets were added, incubated, washed and acquired. The gating was set by forward and side scatter, and 500,000 gated events were acquired. Compensation and gating boundaries were adjusted using unstained, single stained, and Fluorescence Minus One (FMO) control. IFNα and IFNβ levels were measured using VeriKine-Human Interferon Alpha All Subtype and Beta ELISA kits (PBL Interferon sources). IFNλ1, IFNλ2 and IFNλ3 were measured using the Duo Set ELISA kit (R&D Systems).

### Statistical analysis

Cross-sectional analysis of the frequency of dendritic cell subsets and haematology analysis were performed using the polynomial model for the best fit curve (either first-order or second-order model). By way of modeling analysis, first-order or second-order model polynomial function was shown to be the best model amongst the diverse polynomials examined. Mild versus severe statistically significant differences were calculated by the non-parametric Mann–Whitney U test. Flowjo 10.8.0, GraphPad PRISM version 9 (GraphPad Software, Inc.) were used for the data analyses. Correlation analyses were performed using JMP 16 (SAS).

**Table 1:** Demographics and clinical parameters of the study population

Days after RT-PCR confirmation	15–30 days	31–60 days	61–90 days	91–120 days	121–150 days	151–180 days	More than 180 days
Subjects enrolled	n = 46	n = 33	n = 38	n = 34	n = 32	n = 37	n = 40
Median age (range)	41.5 (18–70)	36 (25–68)	45 (19–59)	45 (21–69)	45.5 (27–59)	42 (23–58)	38.5 (21–78)
Gender (M/F)	27/19	17/18	22/15	22/12	14/18	23/16	26/14
Fever, no. (%)	29 (67%)	22 (65%)	28 (74%)	23 (74%)	25 (83%)	23 (72%)	17 (47%)
Chills, no. (%)	9 (21%)	5 (15%)	2 (5%)	7 (22%)	4 (13%)	1 (3%)	3 (8%)
Cough, no. (%)	21 (49%)	20 (59%)	14 (37%)	15 (48%)	14 (47%)	17 (53%)	12 (33%)
Sore throat, no. (%)	21 (49%)	12 (35%)	11 (29%)	12 (38%)	10 (33%)	16 (50%)	13 (36%)
Runny nose, no. (%)	7 (16%)	6 (18%)	5 (13%)	0	3 (10%)	6 (19%)	5 (14%)
Taste loss, no. (%)	24 (55%)	14 (41%)	17 (44%)	12 (39%)	11 (37%)	20 (63%)	12 (33%)
Smell loss, no. (%)	21 (49%)	14 (41%)	21 (55%)	9 (29%)	11 (37%)	16 (50%)	10 (28%)
Muscle aches, no. (%)	23 (53%)	20 (59%)	29 (76%)	15 (48%)	18 (60%)	21 (66%)	13 (36%)
Joint pain, no. (%)	21 (49%)	18 (53%)	20 (53%)	10 (32%)	18 (60%)	14 (44%)	9 (25%)
Abdominal pain, no. (%)	3 (7%)	3 (9%)	4 (11%)	2 (6.5%)	3 (10%)	2 (7%)	3 (8%)
Vomit, no. (%)	3 (7%)	4 (12%)	5 (13%)	4 (13%)	3 (10%)	5 (16%)	3 (8%)
Diarrhea, no. (%)	10 (23%)	5 (15%)	4 (11%)	4 (13%)	6 (30%)	5 (16%)	2 (6%)
Seizures, no. (%)	0	1 (3%)	0	0	0	0	0
Hypertension, no. (%)	11 (26%)	7 (21%)	7 (18%)	7 (23%)	9 (30%)	9 (28%)	8 (22%)
Diabetes, no. (%)	8 (19%)	7 (21%)	11 (30%)	9 (29%)	11 (37%)	8 (25%)	7 (19%)
Asthma, no. (%)	2 (5%)	2 (6%)	1 (3%)	1 ( 3%)	0	1 (3%)	0
Chronic kidney disease, no. (%)	0	0	0	0	1 (3%)	0	1 (3%)
Neuro, no. (%)	0	0	2 (5%)	0	0	0	0
Cancer, no. (%)	0	0	0	0	0	0	0
Heart, no. (%)	1 (6%)	2 (3%)	1 (3%)	0	0	1 (3%)	0
Rheumatic fever, no. (%)	0	0	1 (3%)	0	0	1 (3%)	0
Corticosteroids, no. (%)	4 (9%)	3 ( 9%)	2 (5%)	3 (10%)	1 (3%)	1 (3%)	0
Antiviral drug, no. (%)	4 (9%)	5 (15%)	2 (5%)	4 (13%)	0	0	0
Immunomodulator, no. (%)	0	0	0	0	0	0	0

**Table 2:** Demographics and clinical parameters of the study population

Parameters	Mild	Severe
Subjects enrolled	n = 30	n = 15
Median age (range)	39 (18–81)	48 (22–70)
Gender (M/F)	14/16	12/3
Fever, no. (%)	17 (57)	13 (87)
Chills, no. (%)	4 (13)	6 (40)
Cough, no. (%)	15 (50)	7 (47)
Sore throat, no. (%)	16 (53)	5 (33)
Runny nose, no. (%)	5 (17)	2 (13)
Taste loss, no. (%)	21 (70)	5 (33)
Smell loss, no. (%)	17 (57)	6 (40)
Muscle aches, no. (%)	24 (80)	9 (60)
Joint pain, no. (%)	18 (60)	5 (33)
Abdominal pain, no. (%)	2 (7)	1 (7)
Vomit, no. (%)	2 (7)	1 (7)
Diarrhea, no. (%)	4 (13)	6 (40)
Seizures, no. (%)	NIL	NIL
Hypertension, no. (%)	7 (23)	5 (33)
Diabetes, no. (%)	4 (13)	4 (27)
Asthma, no. (%)	1 (3)	1 (7)
Chronic kidney disease, no. (%)	NIL	NIL
Neuro, no. (%)	NIL	NIL
Heart, no. (%)	NIL	1 (7)
Rheumatic fever, no. (%)	NIL	NIL
Corticosteroids, no. (%)	NIL	3 (20)
Antiviral drug, no. (%)	NIL	4 (27)
Required hospitalization no. (%)	NIL	6 (40)
Required mechanical oxygen support no. (%)	NIL	NIL

## Supplementary Information

**Additional file 1. Fig. S1.** Gating Strategy for DC subsets.

**Additional file 2. Fig. S2.** Analysis of DC subsets, Type I and Type III from acute and convalescent COVID-19 individuals classified as groups based on days since RT-PCR confirmation.

**Additional file 3. Table S1.** 95% of CI of DC subsets and Type I and III IFNs.

## Data Availability

The datasets supporting the conclusions of this article are included within the article and its additional files.
