# Supplementary material for: Restoration of dendritic cell homeostasis and Type I/Type III interferon levels in convalescent COVID-19 individuals
Source: BMC Immunol. 2022 Oct 26;23:51. doi: 10.1186/s12865-022-00526-z (PMC9607715; doi:10.1186/s12865-022-00526-z)
Supplement: Supplementary file 1 — Additional file 1. Fig. S1. Gating Strategy for DC subsets. [file 12865_2022_526_MOESM1_ESM.pdf]

Supplementary Figure.1.

Gating method for Dendritic Cell subsets

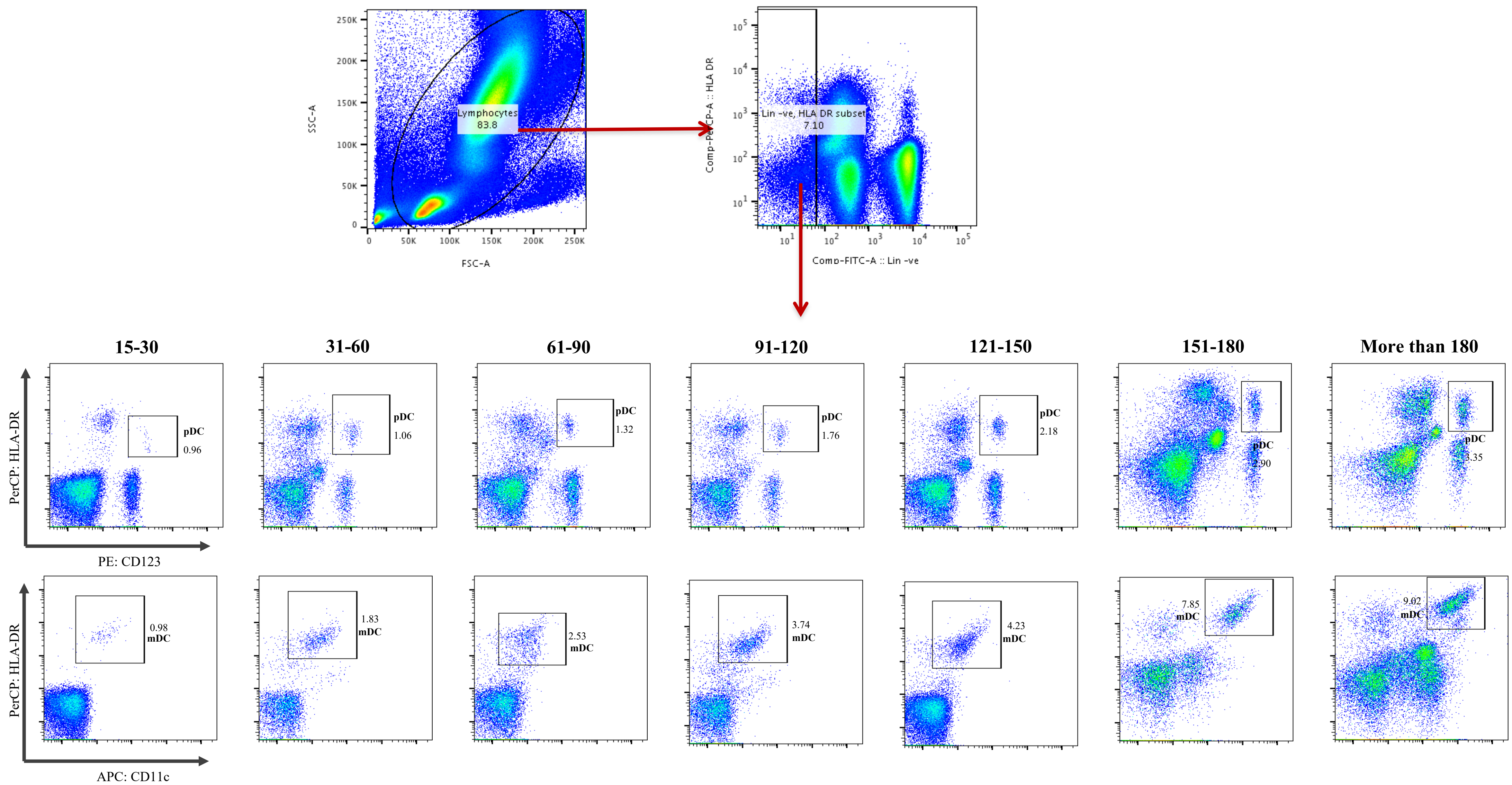

Supplementary Figure 1.

Gating method for DC subsets (A) An illustrative flow cytometry plot from convalescent COVID-19 individual showing the gating method for plasmacytoid (pDC) and myeloid DCs (mDC) Plasmacytoid DC were classified as (Lin– HLA-DR+ CD123+) and myeloid DCs as (Lin– HLA-DR+ CD11c+) classified as groups based on days since RT-PCR confirmation.
