# Supplementary material for: Restoration of dendritic cell homeostasis and Type I/Type III interferon levels in convalescent COVID-19 individuals
Source: BMC Immunol. 2022 Oct 26;23:51. doi: 10.1186/s12865-022-00526-z (PMC9607715; doi:10.1186/s12865-022-00526-z)
Supplement: Supplementary file 2 — Additional file 2. Fig. S2. Analysis of DC subsets, Type I and Type III from acute and convalescent COVID-19 individuals classified as groups based on days since RT-PCR confirmation. [file 12865_2022_526_MOESM2_ESM.pdf]

Supplementary Figure.2

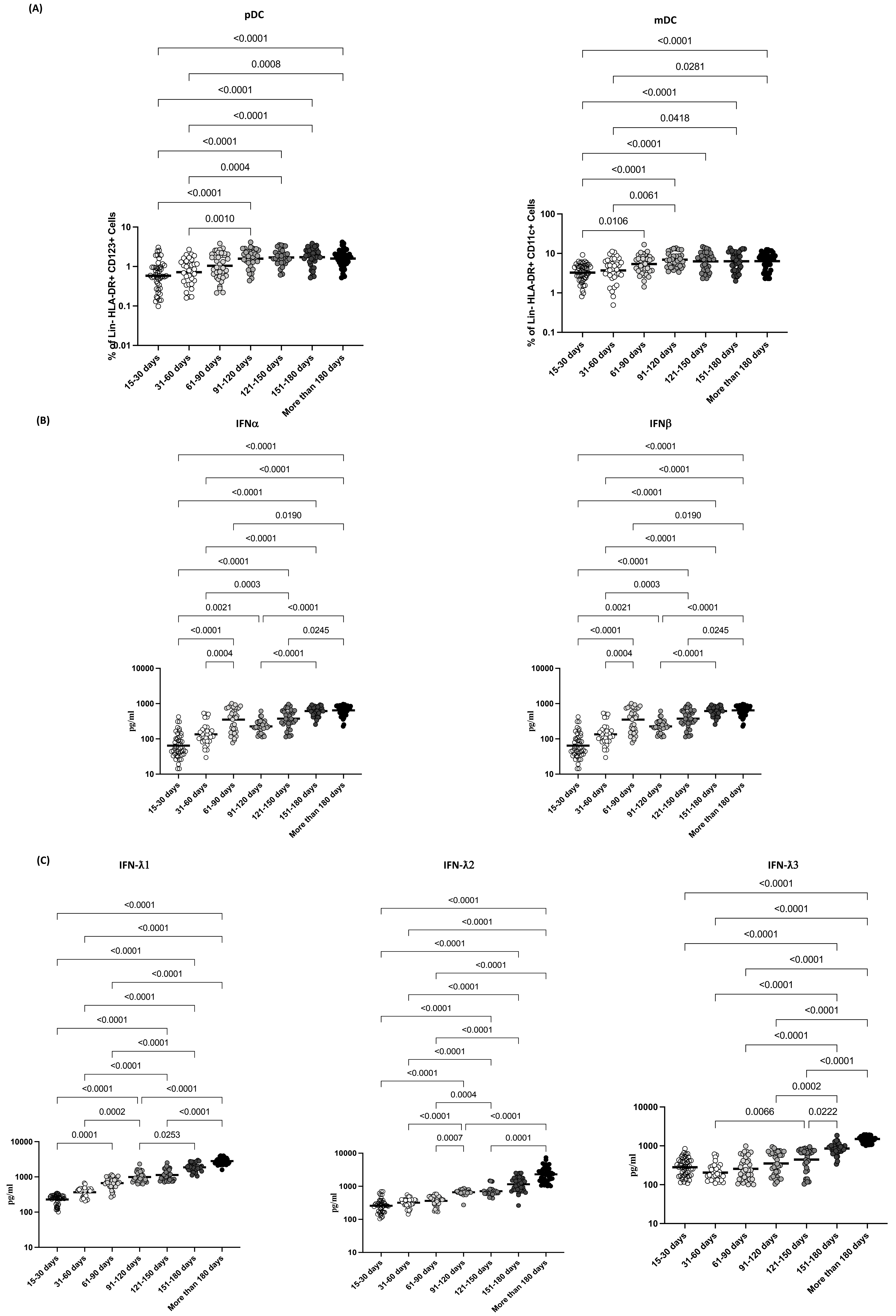

Supplementary Figure 2.

(A). Analysis of DC subsets from acute and convalescent COVID-19 individuals classified as groups based on days since RT-PCR confirmation. The frequencies of DC subsets (pDC and mDC) are shown with a preferred model for the best fit curve. **(B).** Analysis of Type I Interferons (IFNα and IFNβ) from acute and convalescent COVID-19 individuals classified as groups based on days since RT-PCR confirmation. The circulating levels of IFNα and IFNβ are shown with a preferred model for the best fit curve **(C).** Analysis of Type III Interferons (IFNλ1, IFNλ2 and IFNλ3) from convalescent COVID-19 individuals classified as groups based on days since RT-PCR confirmation. The data are represented as scatter plots with each circle representing a single individual. p values were calculated using the Kruskal-Wallis test with Dunn's post-hoc for multiple comparisons.
