# Supplementary material for: Restoration of dendritic cell homeostasis and Type I/Type III interferon levels in convalescent COVID-19 individuals
Source: BMC Immunol. 2022 Oct 26;23:51. doi: 10.1186/s12865-022-00526-z (PMC9607715; doi:10.1186/s12865-022-00526-z)
Supplement: Supplementary file 3 — Additional file 3. Table S1. 95% of CI of DC subsets and Type I and III IFNs. [file 12865_2022_526_MOESM3_ESM.docx]

**Supplementary Table.1. 95% of CI of DC subsets and Type I and III IFNs.**

| S.No | Parameters | 15-30 days | 31-60 days | 61-90 days | 91-120 days | 121-150 days | 151-180 days | More than 180 days | p value |
| --- | --- | --- | --- | --- | --- | --- | --- | --- | --- |
| 1 | pDC | 0.7537 | 0.9505 | 1.341 | 1.918 | 1.995 | 2.060 | 1.859 | <0.0001 |
| 2 | mDC | 3.825 | 4.838 | 6.440 | 8.106 | 7.680 | 7.624 | 7.528 | <0.0001 |
| 3 | IFNα | 81.44 | 172.2 | 455.6 | 260.6 | 457.7 | 684.1 | 710.4 | <0.0001 |
| 4 | IFNβ | 6.701 | 49.21 | 50.16 | 88.32 | 95.04 | 238.9 | 294.8 | <0.0001 |
| 5 | IFNλ1 | 252.6 | 407.4 | 749.3 | 1114 | 1284 | 2037 | 2989 | <0.0001 |
| 6 | IFNλ2 | 296.2 | 356.6 | 406.8 | 706.1 | 794.1 | 1369 | 2681 | <0.0001 |
| 7 | IFNλ3 | 329.1 | 241.7 | 320.1 | 447.1 | 571.0 | 952.0 | 1585 | <0.0001 |
